# Torsional ultrasound modality for hard nucleus phacoemulsification cataract extraction

**DOI:** 10.1136/bjo.2007.128504

**Published:** 2008-06-20

**Authors:** M Zeng, X Liu, Y Liu, Y Xia, L Luo, Z Yuan, Y Zeng, Y Liu

**Affiliations:** 1 Zhongshan Ophthalmic Center, Sun-Yat-Sen University Guangzhou, People’s Republic of China; 2 The Second Affiliated Hospital of Guangzhou Medical College, Guangzhou, People’s Republic of China

## Abstract

**Aim:**

To evaluate the efficacy and safety of phacoemulsification using torsional modality with different parameter settings for hard nucleus cataract extraction.

**Design:**

A prospective, randomised clinical study.

**Methods:**

A clinical practice study conducted at the Cataract Service, Zhongshan Ophthalmic Center, Sun-Yat-Sen University, and Guangzhou. One eye each from 198 consecutive patients with cataract density grade IV according to the Emery–Little system classification system, requiring phacoemulsification and intraocular lens implantation, was included. Eyes were randomly assigned to the Linear Torsional combined with Ultrasound power group (Linear Tor+US group, n = 66), 100% Fixed Torsional group (Fixed Tor group, n = 65) and conventional Ultrasound burst group (US group, n = 67). All surgeries were performed by a single experienced surgeon and outcomes evaluated by another surgeon masked to treatment. Intraoperative parameters were Ultrasound Time (UST), Cumulative Dissipated Energy (CDE) and surgical complications. Patients were examined on post-op days 1, 7 and 30. Postoperative outcomes were final best corrected visual acuity (BCVA), average central and incisional corneal thickness and central endothelial cell counts.

**Results:**

The mean UST was lower in the Fixed Tor group than in the US group and in the Lin US+Tor group (p⩽0.0001). The mean CDE was lower in the Lin Tor+US group and in the Fixed Tor group than in the US group (p⩽0.0001). Comparing with the two Tor group, the US group had a lower average BCVA on post-op 1, 7 (p⩽0.0001) and 30 (p>0.01), greater average central corneal and incisional thickness on days 1, 7 (p⩽0.0001) and 30 (p>0.01), and higher average corneal endothelial cell losses on day 7 and 30 days (p⩽0.0001).

**Conclusions:**

Torsional combined with ultrasound power or high fixed torsional amplitude can yield more effective hard nucleus phacoemulsification than conventional ultrasound modality.

Phacoemulsification followed by intraocular lens (IOL) implantation is a worldwide proven surgical procedure for cataract extraction;^1^ ^2^ consequently, this procedure is being used not only for cataract lens replacements but also for visual corrective or refractive purposes.^3^ ^4^ More ultrasonic energy and time are needed for hard nucleus removal than for softer ones, thus increasing the risk of surgical induced trauma, especially corneal endothelial dysfunction.

The OZil Torsional system (Infiniti, Alcon, Fort Worth, TX) is a hardware and software upgrade which includes a dedicated handpiece that produces side-to-side rotary oscillations of the phaco tip. Comparing with the jackhammer motion in conventional phaco, the improved OZil Torsional oscillation sheers the lens material with virtually no repulsion, thereby dramatically reduced phaco energy required for lens removal without compromising efficiency.^5^ Torsional works at a lower frequency of 32 kHz than the 40∼45 kHz in conventional phaco and theoretically reduces efficiency in lens removal, especially with hard nucleus. We tried different parameter settings for hard nuclear removal, and so far two settings outperformed Linear Torsional amplitude setting alone in efficiency and safety: one was Linear Torsional combined with a short duration of Ultrasound power, and the other was Torsional with fixed high amplitude.

The main aim of this randomised prospective clinical comparative study is to document the potential advantages of Torsional modality with or without ultrasound power setting over conventional Ultrasound modality alone for hard nucleus phacoemulsification. The intraoperative ultrasound energy and time were recorded, and the postoperative visual acuity, central and incisional corneal thickness and corneal endothelial cells count of each case were measured and compared.

## METHODS

One hundred and ninety-eight eyes of 198 consecutive patients having elective phacoemulsification and IOL implantation were included in this prospective study. The average age of the 102 women and 96 men was 69.3 (range 55 to 85). Informed consent was obtained from all the patients before surgery, and the study was conducted in accord with the principles of the Declaration of Helsinki. The study was conducted at the Zhongshan Ophthalmic Center, Guangzhou from May 2006 to February 2007. Patients diagnosed as having age-related cataracts only with a nuclear hardness Grade IV according to the Emery–Little system were recruited. Other inclusive criteria included having a pupil diameter of 7 mm or larger; corneal endothelial cell count greater than 1200/mm^2^; and availability for regular follow-up examinations. Patients were excluded if they had other vision-affecting, visual or systemic disorders—for example, diabetic retinopathy, glaucoma, age-related macular degeneration, uveitis or previous intraocular surgery.

All enrolled subjects underwent standard preoperative examinations to obtain baseline data. All eyes planned for surgery were then randomly assigned to one of the three groups: Linear Torsional in conjunction with the Ultrasound energy group (Linear Tor+US group), 100% Fixed Torsional amplitude group (Fixed Tor group), and the control group using Ultrasound burst mode (US group). The Ultrasound burst model was set a maximum power of 60%; burst width 40 ms; off time 30 ms; vacuum 400 mm Hg, and aspiration 40 cc/min. The parameter settings are shown in table 1.

**Table 1:** Parameters for Fixed Torsional (Fixed Tor) and Linear Torsional+Ultrasound (Lin Tor+US) and Ultrasound groups (US)

Group	Fixed Tor	Lin Tor+US	US
Torsional amplitude	100% (fixed)	100% (linear)	
Ultrasound power (%) (burst)	0	30% (linear)	60
Vacuum limit (mm Hg) (fixed)	400 (fixed)	400 (fixed)	400
Aspiration flow rate (cm^3^/min) (fixed)	40 (fixed)	40 (fixed)	40

All surgery was performed by the same experienced surgeon (YL) with a standard quick chop technique^6^ using Alcon Infiniti System (Alcon, Fort Worth, TX). For all groups, a MicroTip 0.9 mm ABS phaco tip (45°, Kelman) with Microsmooth High Infusion Sleeve was used. All the operations used a standard setting. All patients received topical anaesthesia of 0.5% proparacaine hydrochloride eye-drops (Alcaine) before surgery. A 3.2 mm wide self-sealing temporal clear corneal incision was made on the temporal side of the eye. DuoVisc and soft-shell techniques were used to reform and stabilise the surgical planes and protect the corneal endothelium.^7^ A 5.5–6.0 mm continuous curvilinear capsulorhexis was performed with a 26-gauge needle. All intraocular lenses were inserted into capsular bag with the same injector system. No suture of the incision was needed at the end of surgery.

The main system parameters were ultrasound time (UST), and cumulative dissipated energy (CDE). US time represents the total time in seconds that U/S (or OZil) remained active. CDE correlates to the total amount of energy at the incision. CDE is calculated as follows for Phaco: CDE = average U/S power×U/S time. In Torsional mode, the CDE was calculated as: Torsional amplitude×Torsional time×0.4. The frequency of the phaco tip in Torsional mode was 80% of the standard phaco (32 kHz in Torsional versus 40 kHz in Phaco), and the travel distance of phaco tip in Torsional mode was half that in standard phaco. This helped justify setting the coefficient to 0.4. The UST and CDE values in Torsional and Phaco mode were automatically calculated and displayed on the monitor of the phaco system.

**Figure 1:**
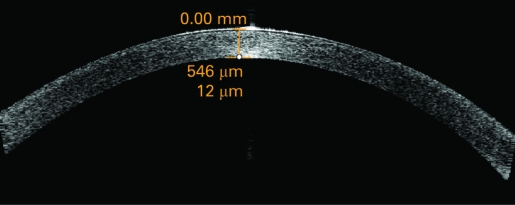
Central corneal thickness as measured and shown by anterior segment imaging VISANTE optical coherence tomography before operation.

**Figure 2:**
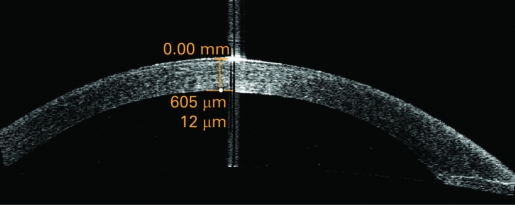
Central corneal thickness as measured and shown by anterior segment imaging VISANTE optical coherence tomography 1 day after operation.

**Figure 3:**
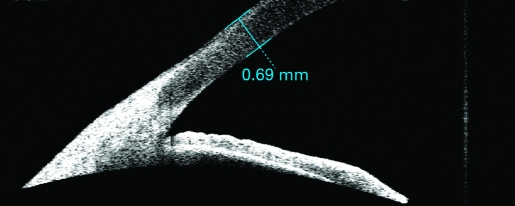
Peripheral corneal thickness as measured and shown by anterior segment imaging VISANTE OCT before operation.

**Figure 4:**
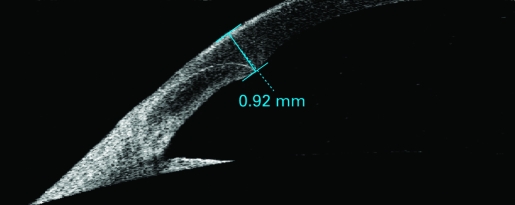
Incisional corneal thickness as measured and shown by Anterior Segment Imaging VISANTE OCT 1 day after operation.

Postoperative outcomes were assessed by another ophthalmologist (ZM) who was masked to the treatment assignment. Patients were examined on post-op days 1, 7 and 30. The postoperative best corrected visual acuity (BCVA) and complications were documented. The central corneal thickness was measured using the 180° single scan through the central light reflecting point of the pupil (figs 1, 2) with the Anterior Segment Imaging VISANTE OCT 1000 (Carl Zeiss, Vertrieb Deutschland, Germany). The incisional corneal thickness was measured using the single scan through central light reflecting point of pupil and the middle of the incision (figs 3, 4).^8^ ^9^ The endothelial cell counts were measured using the non-contact special microscope (SP-2000 P, Topcon, Tokyo). More than 100 endothelial cells per eye were used to calculate the cell density using the IMAGEnet 2000, version 2.53 software (Topcon). At each of these visits, three photographs per eye were taken. The mean of the three results was used to represent this outcome.

SPSS 13.0 for Windows XP software (SPSS, Chicago) was used to test the difference in UST, CDE count, logMAR visual acuity, central corneal thickness and endothelial cell counts with one-way ANOVA and LSD posthoc multiple comparison. Two-sided testing was performed with alpha set at 0.01.

## RESULTS

A total of 198 eyes were enrolled in the study, of which 66 were in the Linear Torsional+linear Ultrasound group, 65 were in the Fixed torsional group, and 67 were in the Ultrasound group.

One-way ANOVA and multiple comparisons of mean UST showed statistically significant differences (p⩽0.0001) between each two groups (p⩽0.0001), with the longest UST in US group and the shortest in Fix Tor group. Multiple comparison of mean CDE showed a significantly higher CDE in the US group than in the two Tor groups (p⩽0.0001, p⩽0.0001 respectively) but no significant difference between the two Tor groups (p = 0.196) (table 2).

**Table 2:** Ultrasound time (UST) and cumulative dissipated energy (CDE) by group

Group	UST	CDE
Lin Tor+US (n = 66) (SD)	47.77 (17.23)	15.89 (6.86)
Fixed Tor (n = 65) (SD)	42.41 (16.05)	15.51 (5.59)
US (n = 67) (SD)	61.01 (18.84)	17.43 (7.21)
F value*	146.49	46.889
p Value	⩽0.0001	⩽0.0001

*One-way ANOVA.

Fixed Tor, Fixed Torsional amplitude group; Lin Tor+US, Linear Torsional combined with Ultrasound energy; US, Ultrasound mode.

There were no cases of posterior capsular rupture in either of the three groups. There was one case of incisional burn in the US group. No postoperative complications such as fibrin formation, synechias, macrophages on the IOL optic, or endophthalmitis were observed in any patient at least during this short-term follow-up.

One-way ANOVA and multiple comparison of BCVA showed a statistically significantly better BCVA in two Tor groups than in the US group on post-op day 1 (p⩽0.0001, p⩽0.0001) and day 7 (p⩽0.0001, p⩽0.0001), but no significant differences between the two Tor groups on the same post-op day (p = 0.724, p = 0.231) and no significant difference among the three groups on post-op day 30 (table 3).

**Table 3:** Best corrected visual acuity by group at 1, 7 and 30 days

Group	Follow-up visits (days)		
	1	7	30
Lin Tor+US (SD)	0.18 (0.10)	0.027 (0.014)	−0.075 (0.01)
Fixed Tor (SD)	0.19 (0.08)	0.028 (0.016)	−0.077 (0.01)
US (SD)	0.22 (0.11)	0.083 (0.015)	−0.070 (0.02)
F value*	18.996	138.44	0.433
p Value	⩽0.0001	⩽0.0001	0.650

*One-way ANOVA.

Fixed Tor, Fixed Torsional amplitude group; Lin Tor+US, Linear Torsional combined with Ultrasound energy; US, Ultrasound mode.

The difference of baseline central and peripheral corneal thickness was not significant between groups (p = 0.824). The central and incisional corneal thickness were significantly greater in US group than in the two Tor group on post-op day 1 (p⩽0.0001) and day 7 (p⩽0.0001), while the difference between the two Tor groups was not significant on the same post-op days (p>0.01), and there was no significant difference among the three groups on post-op day 30 (p = 0.732) (tables 4 and 5).

**Table 4:** Mean central corneal thickness change by group

Group	Lin Tor+US		Fixed Tor		US		F*	p Value
	Corneal thickness	Change, %	Corneal thickness	Change, %	Corneal thickness	Change, %		
Preop (SD)	531 (21)		535 (26)		539 (34)		0.194	0.824
1 day (SD)	617 (48)	96 (8), 18.1	621 (54)	96 (11), 17.9	633 (60)	104 (12), 19.3	47.362	<0.0001
7 days (SD)	565 (25)	44 (10), 8.3	568 (32)	43 (10), 8.03	587 (38)	58 (10), 10.7	57.718	<0.0001
30 days (SD)	534 (22)	6 (5), 1.1	535 (26)	−6 (5), −1.1	532 (20)	−7 (4), −1.3	0.345	0.732

*One-way ANOVA.

Fixed Tor, Fixed high Torsional amplitude group; Lin Tor+US, Linear Torsional combined with Ultrasound energy; US, Ultrasound mode.

**Table 5:** Mean incisional corneal thickness change by group

Group	Lin Tor+US		Fixed Tor		US		*F	p Value
	Corneal thickness	Change, %	Corneal thickness	Change, %	Corneal thickness	Change, %		
Preop (SD)	699 (28)		687 (31)		703 (36)	0.916	0.363	
1 day (SD)	919 (47)	220 (29), 31.5	903 (54)	216 (21), 31.4	1058 (44)	355 (15), 50.5	96.582	<0.0001
7 days (SD)	871 (28)	181 (25), 25.9	865 (42)	178 (20), 25.9	936 (36)	234 (13), 32.3	85.374	<0.0001
30 days (SD)	703 (24)	4 (3), 0.53	695 (31)	8 (5), 1.1	708 (26)	5 (3), 0.71	1.148	0.256

*One-way ANOVA.

Fixed Tor, Fixed high Torsional amplitude group; Lin Tor+US, Linear Torsional combined with Ultrasound energy; US, Ultrasound mode.

The difference in central endothelial corneal cells counts among all the groups was not significant before surgery (p = 0.351), but it was significant 7 days and 30 days after surgery. The US group caused more central endothelial cell losses than the other two groups. The difference of average endothelial cells between the US group with Lin Tor+US and with Fixed Tor group was statistically significant (p⩽0.0001 respectively), but the difference between Lin Tor+US and Fixed Torsional is not significant (p>0.01) (table 6).

**Table 6:** Mean endothelial corneal cell counts (ETC) count by group

Group	Lin Tor+US		Fixed Tor		US		F*	p Value
	ETC	ETC change, %	ETC	ETC change, %	ETC	ETC change, %		
Preop (SD)	2450 (298)		2431 (287)		2418 (283)	0.944	0.351	
7 days (SD)	2297 (322)	−153 (132), −6.2	2277 (298)	−154 (109), 6.3	2132 (331)	−286 (106), −11.8	2.982	0.004
30 days (SD)	2191 (343)	−259 (185), −10.6	2176 (320)	−255 (171), 10.5	2089 (349)	−329 (150), −13.6	3.346	0.001

*One-way ANOVA.

Fixed Tor, Fixed high Torsional amplitude group; Lin Tor+US, Linear Torsional combined with Ultrasound energy; US, Ultrasound mode.

## DISCUSSION

Recent developments in phacoemulsification have made cataract removal safer and more efficient. Technological advances have provided more options, allowing surgeons to customise their techniques, to reduce phacoenergy and duration. However, phacoenergy is still the main risk factor for surgical induced trauma, especially for corneal endothelial cell injury or dysfunction. Phacoemulsification advances aim to reduce the phacoenergy and shorten the phacotime.^10^ ^11^

In traditional phaco, the longitudinal movement of the phaco tip tends to push the nuclear away with each forward stroke, so the ultrasound has to be purposely interrupted to reattract the nuclear fragment to the tip; furthermore, only the forward stroke has cutting effect. While in Torsional phaco, although the tip moves at a lower frequency of 32 kHz than the 40 kHz in traditional phaco, the side-to-side tip movement sheers the lens material with no repellent force, and cuts with both direction of the tip movement, thus significantly improving emulsify efficiency.

NeoSoniX employs a similar rotational oscillation to Torsional but at a much lower frequency of 100 Hz, thereby compromising its emulsify efficiency, especially in the case of a dense nucleus.^12^ ^13^ Ozil Torsional Technology provides the flexibility of being used alone or in combination with different levels of standard high-frequency Ultrasound energy for different lens densities. One of the main purposes of this study was to evaluate the efficiency of Torsional with or without Ultrasound power in handling a hard nucleus, and Torsional was also compared with the proven energy effectively economised modality Ultrasound Burst mode. Torsional amplitude was preset at 100% with minimal additional phaco power in order to reduce the total energy delivered into the eye. In this combined mode, the working time of Torsional over ultrasound was 4:1 in each cycle. From our experiences, most nuclei of various density can be emulsified using Linear Torsional Amplitude 100% combined with at most 30% Ultrasound energy, and the softer the nucleus, the less ultrasound energy required.

Torsional Amplitude can also be be set at Fixed Amplitude with no US. In the present study, Torsional amplitude was fixed at 100%, which means that the Torsional amplitude reached 100% once the pedal was in the third position. The Fixed Amplitude setting and lineal setting are easily interchangeable on the touch screen. With linear setting, although one could also attain 100% by pressing the pedal to the deepest point, surgeons are usually hesitant to go this far and end up applying less amplitude than may be necessary. Another option is setting the treadle of foot position 3 to about 10% to reach maximum amplitude, but it it has to be reset when high Torsional amplitude is not necessary for the remaining soft nucleus. Less frictional movements within the incisional and lower frequency used in Torsional modality reduced the risk for thermal injury, thus making it safe even with 100% Fixed Amplitude, as was shown in our study in which no cases of incisional burn were found. While in conventional mode, the risks of incisional burn increased with the level of phaco power applied.^14^

In the present study, the vacuum was set at 400 mm Hg and aspiration rate at 40 cm^3^/min, settings made possible by the improved Fluidics Management System,^15^ which has a low-compliance tubing and cassette. The advantage of using a higher vacuum and aspiration rate, a technique known as ultrasound-assisted phacoaspiration, makes the whole surgical process less invasive, reducing surgical time and energy. An appropriate phaco tip is another important factor influencing efficient emulsification. In our study, a 0.9 mm MicroTip ABS phaco tip (45°, Kelman) with Microsmooth High Infusion Sleeve was used, although the 0.9 mm Tapered ABS phaco tip (45°, Kelman) is recommended for the soft nucleus because it holds the fragment better than the Microtip ABS. In the case of a hard nucleus, it is easily occluded by the nucleus fragments and needs to be manually rinsed.^16^ ^17^

Intraoperative parameters of UST and CDE were compared between groups. There was a significant difference in UST among the three groups, with the longest UST in US group and the shortest in the Lin Tor+US group. And as for CDE, it was significantly higher in the US group than the two Tor groups, while no significant difference was found between the two Tor groups. These data suggested that conventional modality was more time- and energy-consuming.

The anterior segment OCT is a novel corneal pachymetry instrument, which can be used for precise measurement of central and incisional corneal thickness with a high resolution. The central and incisional corneal thickness and corneal endothelial cells loss were indicators for surgical induced corneal trauma.^18^ ^19^ In our study, CDE in the Tor+US, fixed Tor and US groups was 15.89, 15.51 and 17.43, respectively, and the average endothelial cell losses were 259 (10.6%), 255 (10.5%) and 329 (13.6%) accordingly. This finding is consistent with other reports where endothelial cells loss was correlated with ultrasound energy applied.^20^ ^21^ Since all surgeries were performed by the same experienced surgeon using the same techniques and settings in our study, variations due to surgical techniques were minimised, and the disparity in corneal injury more likely resulted from different energy settings. Corneal thickness returned to baseline 1 month after surgery while endothelial loss persisted. This could be explained by the compensation of the remaining endothelial cells.

Another concern was whether the difference in energy delivery would be of clinical significant in eyes using the combined modality. The average post-op BCVA was significantly better in the Lin Tor+US group and Fixed group than in the US group on days 1 and 7, but this advantage did not remain 1 month after surgery. This suggests that the Lin Tor+US group and Fixed Tor group tend to produce a better visual outcome in the early postoperative phase. This pattern of visual rehabilitation after surgery is likely attributable to corneal injury and its recovery. Almost all patients recovered 7 days after surgery, though some suffered from mild corneal oedema. The safety of Torsional may be of paramount importance in high-risk cases with low endothelial cell counts or when surgery is performed by a less experienced surgeon.

Our results show that Torsional combined with ultrasound power and Fixed high Torsional amplitude are both effective and safe for hard nucleus extraction with less UST and CDE, thus causing less corneal injury and promoting earlier visual acuity recovery than the conventional modality.
